# TRPC6 and TRPC4 Heteromultimerization Mediates Store Depletion-Activated NCX1 Reversal in Proliferative Vascular Smooth Muscle Cells

**DOI:** 10.1080/19336950.2018.1451696

**Published:** 2018-04-10

**Authors:** Bin Zhang, Bei Liu, Carolyn M. Roos, Michael A. Thompson, Y. S. Prakash, Jordan D. Miller, Rui-wei Guo

**Affiliations:** a Division of Cardiovascular Surgery, Mayo Clinic, Rochester, MN, USA; and Department of Physiology, Mayo Clinic, Rochester, MN, USA; b Department of Obstetrics and Gynecology, Kunming General Hospital of Chengdu Military Command, Kunming, Yunnan, China; c Department of Anesthesiology, Mayo Clinic, Rochester, Minnesota, USA; d Department of Cardiology, Kunming General Hospital of Chengdu Military Command, Kunming, Yunnan, China

**Keywords:** transient receptor potential canonical, store-operated calcium channels, Na^+^/Ca^2+^ exchange, vascular smooth muscle cells

## Abstract

Store depletion has been shown to induce Ca^2+^ entry by Na+/Ca+ exchange (NCX) 1 reversal in proliferative vascular smooth muscle cells (VSMCs). The study objective was to investigate the role of transient receptor potential canonical (TRPC) channels in store depletion and NCX1 reversal in proliferative VSMCs. In cultured VSMCs, expressing TRPC1, TRPC4, and TRPC6, the removal of extracellular Na^+^ was followed by a significant increase of cytosolic Ca^2+^ concentration that was inhibited by KBR, a selective NCX1 inhibitor. TRPC1 knockdown significantly suppressed store-operated, channel-mediated Ca^2+^ entry, but TRPC4 knockdown and TRPC6 knockdown had no effect. Separate knockdown of TRPC1, TRPC4, or TRPC6 did not have a significant effect on thapsigargin-initiated Na^+^ increase in the peripheral regions with KBR treatment, but knockdown of both TRPC4 and TRPC6 did. Stromal interaction molecule (STIM)1 knockdown significantly reduced TRPC4 and TRPC6 binding. The results demonstrated that TRPC4–TRPC6 heteromultimerization linked Ca^2+^ store depletion and STIM1 accumulation with NCX reversal in proliferative VSMCs.

## 1. Introduction

The regulation of intracellular calcium concentration ([Ca^2+^]i is an important control of vascular smooth muscle cell (VSMC) proliferation [1]. Currently known regulators of [Ca^2+^] in VSMCs include Na^+^/Ca^2+^ exchange (NCX), voltage-operated Ca^2+^ channels, and receptor-operated Ca^2+^ channels (ROC) in the plasma membrane (PM), which include second messenger-gated channels, ionotropic receptors, and store-operated calcium channels (SOCC) [2,3]. Ca^2+^ entry mediated by SOCC and NCX1 reversal-mediated Ca^2+^ entry are both known to influence VSMC proliferation and NCX1 is also involved in the migration and proliferation of pulmonary artery smooth muscle, gastrointestinal myofibroblast, and fibroblast cell lines [5–7].

STIM1, a Ca^2+^ sensor located in the endoplasmic reticulum (ER), and Orai1, a Ca^2+^ channel present in the PM, have been identified as molecular components of SOCCs [4,8]. When Ca^2+^ in the ER store is depleted, STIM1 relocates to the PM and combines with calcium release-activated calcium modulator 1 (ORAI1) which mediates Ca^2+^ entry. NCX1 is a PM ion transporter expressed in the PM of VSMCs [9] that usually acts in forward mode to transport Ca^2+^ out of cells. In some pathological conditions it acts in reverse mode, i.e. NCX reversal, to transport Ca^2+^ into cells. The direction of transport depends on the Na^+^ and Ca^2+^ concentration gradients [10].

We previously showed that store depletion-induced Ca^2+^ entry by NCX1 reversal in VSMCs, but did not investigate the mechanism. However, members of the transient receptor potential canonical (TRPC) family are expressed in the PM of VSMCs and have been proposed as mediators of nonselective store-operated ion entry [11]. Consequently, we hypothesized that store depletion may induce TRPCs-mediated Na^+^ entry and lead to NCX1 reversal. TRPC–NCX coupling has been observed with TRPC3 and NCX in HEK-293 human embryonic kidney cells and rat cardiomyocytes [12,13]. Poburko et al. showed that TRPC6 mediated agonist-induced [Na^+^]i transients in close proximity of the PM that stimulated NCX reversal and enhanced Ca^2+^ entry in the A7r5 VSMC cell line [14]. As TRPCs respond differently to store depletion in different cell lines, the study objective was to investigate the their involvement in store depletion and NCX1 reversal in VSMCs.

## 2. Methods

The reagents and solutions used in the study are described in detail in the supplementary information available online. The experimental procedures used for VSMC dispersion and culture, cell transfection, Na^+^ and Ca^2+^ measurements, coimmunoprecipitation, and other procedures are described in the Data Supplement.

## 3. Results

### 3.1. NCX1 reversal was activated by store depletion and TRPC1, TRPC4, and TRPC6 expression in primary cultures of proliferative aortic VSMCs

**Figure 1.**
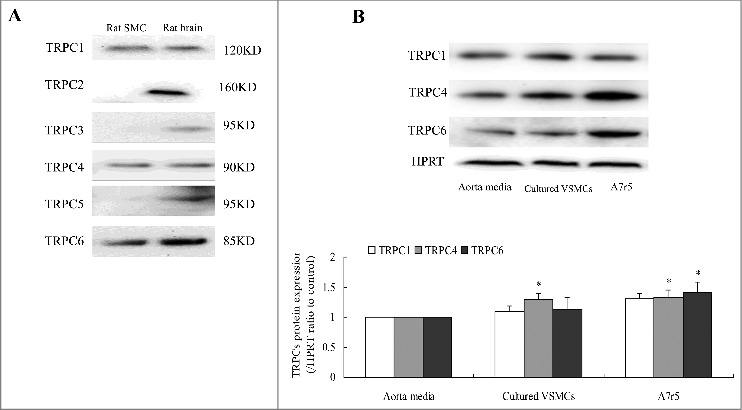
TRPCs subunit expression was assayed in proliferative VSMCs. (A) Western blots of TRPC1, TRPC4, and TRPC6 protein expression in primary cultures of rat aorta VSMCs. Rat brain was used as a positive control. (B) TRPC4 protein expression was higher in primary cultures of aortic VSMCs and in A7r5 cells than in freshly isolated VSMCs from aorta media. TRPC6 protein expression were higher in only A7r5 cells. n = 6. *P < 0.05 vs. control.

We previously described NCX1 regulation of Ca^2+^ entry in VSMCs that was functionally associated with store depletion (Supplemental Figure I). Ca^2+^ entry mediated by NCX1 reversal depends on [Na^+^]i transients, and store depletion can activate TRPCs to increase Na^+^ entry. Expression of TRPC1, TRPC4, and TRPC6 in primary cultures of proliferative aortic VSMCs was assayed to investigate the molecular mediators of store depletion-activated NCX1 reversal. The study results revealed which TRPCs were involved. A primary culture of rat aortic VSMCs was established, and immunofluorescence staining with antibody against rat smooth muscle-actin alpha demonstrated that the purity of cultured SMCs was >98% (Supplemental Figure II). Western blot assays showed that TRPC1, TRPC4, and TRPC6 were expressed in cultured VSMCs (Fig. 1A). TRPC4 protein expression was higher in primary cultures of proliferative aortic VSMCs and cultured A7r5 cells than in VSMCs freshly isolated from the aortic media. TRPC6 protein expression was increased in only the A7r5 cells (Fig. 1B).

### 3.3. TRPC1 was involved in store depletion-induced Ca^2+^, but not Na^+^, entry

**Figure 2.**
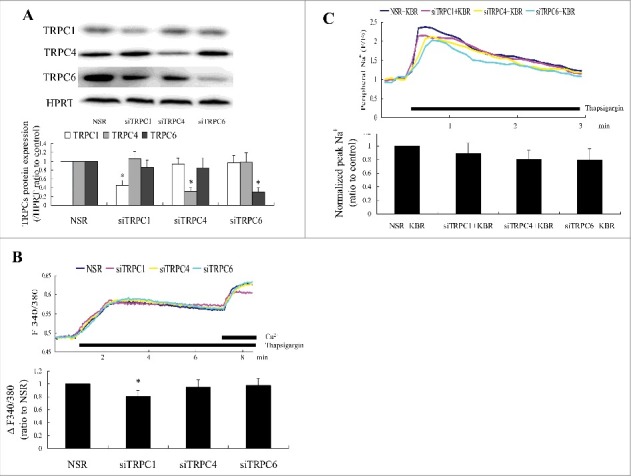
The effect of TRPCs knockdown on store depletion-induced Ca^2+^ and Na^+^ entry was determined in primary cultures of rat aorta VSMCs. (A) TRPC1, TRPC4, and TRPC6 were assayed 72 h after siRNA transfection. siRNA knockdown was target-protein specific, and had no effect on other TRPCs. n = 6. *P < 0.05 vs. nonsilencing RNA (NSR) controls. (B), TRPC1 knockdown, but not TRPC4 and TRPC6 knockdown, decreased store depletion-induced Ca^2+^ entry. Twelve cells and six independent experiments were evaluated. *P < 0.05 vs. NSR. (C) The separate knockdown of TRPC1, 4, and 6 had no effect on thapsigargin-stimulated increase of external Na^+^ concentration peripheral to VSMC treated with KBR. Twelve cells and six independent experiments were evaluated. *P < 0.05 vs. NSR.

To investigate the involvement of TRPC homologues in store depletion-mediated Ca^2+^ and Na^+^ entry, siRNAs were used to knock down TRPC1, 4, and 6 expressions. siRNA transfection significantly downregulated the corresponding TRPCs without detectable changes in the expression of the other isoforms (Fig. 2A). Consistent with previous studies, Ca^2+^ and Na^+^ entry were detected in TRPC1, TRPC4, and TRPC6 knockdown cells. TRPC1 knockdown significantly inhibited Ca^2+^ entry, but neither TRPC4 nor TRPC6 knockdown had an effect on Ca^2+^ entry (Fig. 2B). Individual knockdown of TRPC1, TRPC4, and TRPC6 did not significantly influence the thapsigargin-initiated [Na^+^] increase in the peripheral regions observed in KBR-treated cultures (Fig. 2C). The results demonstrated that TRPC1 was involved in store-operated calcium channel function, but was not required for store depletion-mediated Na^+^ entry.

### 3.4. TRPC6–TRPC4 heteromultimerization mediated store depletion-activated NCX1 reversal

**Figure 3.**
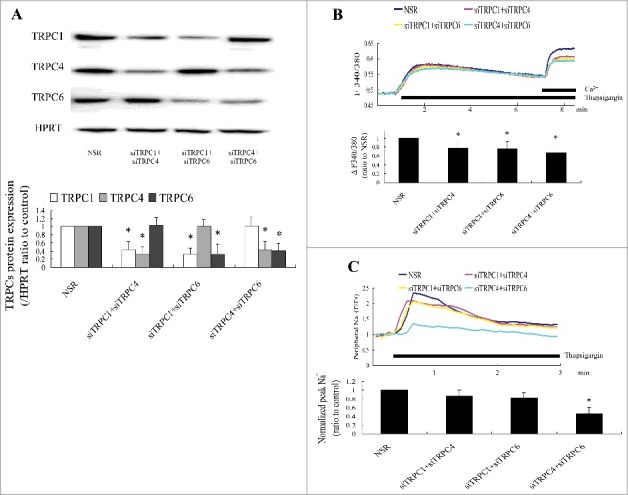
Simultaneous TRPC4 and TRPC6 knockdown deceased store depletion-induced Na+ entry in primary cultures of rat aorta VSMCs. (A) Two different TRPCs were knocked down by transfection with two specific siRNA probes targeting TRPC1, TRPC4, or TRPC6 and assayed 72 h after transfection. n = 6. *P < 0.05 vs. nonspecific siRNA (NSR). (B) Knockdown of two different TRPCs decreased store depletion-induced Ca^2+^ entry. Twelve cells and six independent experiments were evaluated. *P < 0.05 vs. nonsilencing RNA controls. (C) Knockdown of TRPC4 and 6 suppressed thapsigargin-stimulated increase of external Na^+^ concentration peripheral to VSMC treated with KBR. Twelve cells and six independent experiments were evaluated. *P < 0.05 vs. NSR. siTRPC: TRPC-specific siRNA.

Interaction of TRPC3 and TRPC6 with NCX proteins has been shown to mediate ROC and NCX reversal in rat cardiac myocytes and A7r5 cells. TRPC6 activity is regulated indirectly by STIM1 via heteromultimerization with TRPC4. We therefore cotransfected cells with two siRNA probes targeting TRPC1, TRPC4, or TRPC6 to arbitrarily knock down two TRPCs together without affecting the other isoform (Fig. 3A). Simultaneous knockdown of two TRPCs significantly attenuated store depletion-induced Ca^2+^ entry (Fig. 3B), but only TRPC4 and TRPC6 knockdown inhibited thapsigargin-initiated increase in [Na^+^] in the peripheral regions. KBR-treated cultures (Fig. 3C). The results indicated that endogenous TRPC4–TRPC6 heteromultimerization may have been involved in store depletion-activated NCX1 reversal.

### 3.5. TRPC6–TRPC4 heteromultimerization required STIM1

**Figure 4.**
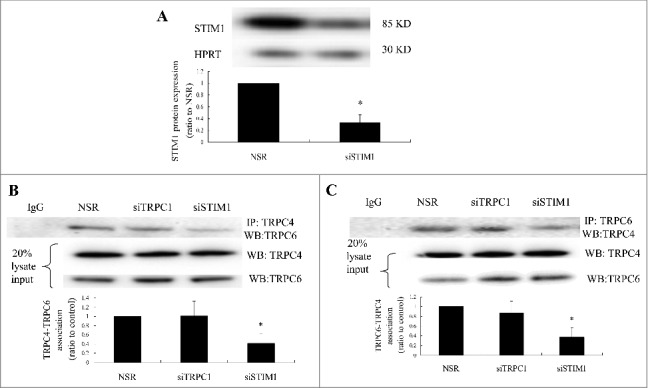
TRPC4–TRPC6 interaction in store depletion-mediated NCX1 reversal. (A) The siRNA against rat STIM1 decreased STIM1 expression by 0.31-fold at 72 h after transfection. n = 6.*P < 0.05 vs. nonsilencing RNA. (B) Cells transfected with siTRPC1 and siSTIM1 for 72 h were treated with thapsigargin (1 µM) for 5 min. TRPC4 and TRPC6 in cell lysates were coimmunoprecipitated. STIM1 knockdown decreased TRPC4 and TRPC6 coprecipitation compared with transfection of nonsilencing RNA and TRPC1-specific siTRPC1. (C) Cells were treated as above and TRPC6 and TRPC4 were coprecipitated. STIM1 knockdown decreased the coprecipitation of TRPC6 and TRPC4 association. n = 6. *P < 0.05 vs. NSR and siTRPC1. Twenty percent of the cell lysates were loaded and served as controls. IgG was a negative control.

**Figure 5.**
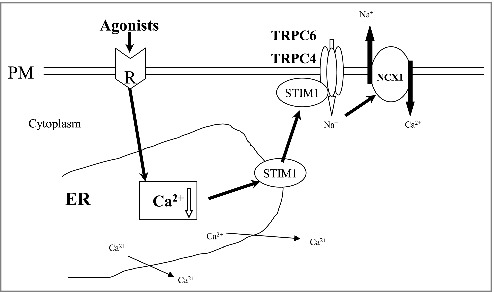
Schematic model of store depletion-activated NCX1 reversal in proliferative vascular smooth muscle cells mediated by TRPC6 and TRPC4 heteromultimerization.

To confirm the interaction of TRPC6 and TRPC4, the effect of STIM1 knockdown on TRPC4–TRPC6 heteromultimerization was investigated in VSMC cultures. siRNA-mediated silencing of STIM1 (siSTIM1) downregulated STIM1 expression by 0.31-fold at 72 h after transfection (Fig. 4A). Coimmunoprecipitation of TRPC6 and TRPC4 was observed, and STIM1 knockdown significantly reduced TRPC4 and TRPC6 binding. Nonspecific binding was assayed using anti-rabbit IgG, which failed to pull down TRPC4–TRPC6 (Fig. 4B, C). The observations suggest that heteromultimerization of TRPC6 and TRPC4 regulated by STIM1 mediated store depletion-activated NCX1 reversal in primary cultured VSMCs (Fig. 5).

## 4. Discussion

This study showed for the first time that TRPC6–TRPC4 heteromultimerization mediated store depletion-activated NCX1 reversal in proliferative VSMCs and that heteromultimerization required STIM1. This conclusion is supported by several lines of evidence. First, NCX1 reversal was activated by store depletion in proliferative VSMCs. Second, TRPC1, TRPC4, and TRPC6 were expressed in primary cultures of aortic VSMCs. Third, simultaneous TRPC4 and TRPC6 knockdown inhibited store depletion-mediated NCX1 reversal. Fourth, STIM1 knockdown significantly reduced TRPC4 and TRPC6 binding. Store depletion thus induced Na^+^ entry via TRPC6–TRPC4 heteromultimerization with activation of NCX1 reversal in proliferative VSMCs.

An aim was to identify the TRPC isoform that mediated SOCC activation and NCX1 reversal. As shown previously [14], TRPC1, TRPC4, and TRPC6 proteins were expressed in the VSMCs used in this study. TRPC1 knockdown significantly suppressed store-operated, channel-mediated Ca^2+^ entry, but had no significant effect on thapsigargin-initiated Na^+^ increase in the peripheral regions in cultures treated with KBR. The results indicated that TRPC1 activity was important for the function of store-operated Ca^2+^ channels and that TRPC6 or TRPC4 knockdown did not inhibit thapsigargin-induced Ca^2+^ and Na^+^ entry. Soboloff et al. showed that TRPC6 knockdown had no significant effect on the amount of Ca^2+^ released by thapsigargin or on store-operated channel-mediated Ca^2+^ entry [15]. However, TRPC6 siRNA has been found to significantly reduce phospholipase C-coupled vasopressin receptor activation-initiated store depletion-mediated Ca^2+^ entry [16]. Even if the complete emptying of Ca^2+^ stores affected by thapsigargin is not a normal physiological event [15], the results indicate that the TRPC6 and TRPC4 channels play a minor role in purely store-operated Ca^2+^ entry in proliferative VSMCs.

Functional associations of TRPC3 and NCX in HEK-293 cells and cardiomyocytes, and TRPC6 and NCX in A7r5 cells have been reported [12–14], but TRPC6 or TRPC4 knockdown did not inhibit thapsigargin-initiated Na^+^ elevation in the peripheral regions. This result was not expected because most functional TRPC channels are heterotetrameric complexes of different TRPC subunits. Yuan et al. reported that the store depletion sensor STIM1 regulated TRPC6 function indirectly by mediating the heteromultimerization of TRPC6 and TRPC4 [17]. In the present study, simultaneous TRPC4 and TRPC6 knockdown inhibited thapsigargin-initiated Na^+^ elevation in the peripheral regions by KB-R7943 treatment, indicating that endogenous TRPC4–TRPC6 heteromultimerization was involved in store depletion-activated NCX1 reversal. However, Figures 3 show the lack of effect of TRPC6 or TRPC4 knockdown alone on thapsigargin-initiated [Na^+^]i transients. Current evidence indicates that even after a substantial reduction of channel protein by interfering RNA, TRPC6 and TRPC4 are still effective in activating NCX reversal, and the function of TRPC4–TRPC6 heteromultimerization was changed only after simultaneous TRPC6 and TRPC4 knockdown [18]. Actually, It remains unclear that if TRPC4-6 heteromultimeric channels are required for Na^+^ influx that siRNA knockdown of the individual subunits has no effect, the knockout model of TRPC6 or TRPC4 should been used to study the deeply mechanism. Some studies suggest that Ca^2+^ influx mediated by NCX is the primary mechanism of Ca^2+^ entry in ATP-stimulated A7r5 cells and requires functional coupling with TRPC6 nonselective cation channels [14,15,19]. In the present study, TRPC4–TRPC6 heteromultimerization mediated store depletion-induced NCX reversal. Therefore, while ATP may operate solely through TRPC6 homomers, store depletion may operate via TRPC6 heteromers. The results of coimmunoprecipitation of TRPC6 and TRPC4 provide some support for that. TRPC function is STIM1-independent at a low STIM1/TRPC channel ratio, but at a high STIM1/TRPC channel ratio, the channels are opened by the interaction of positively charged STIM1 with negative charges in the C-terminal domain of TRPC channels [17,20]. The study results showed that TRPC6 channels were predominant in A7r5 cells and more strongly expressed than in proliferative VSMCs. That might explain the difference in the response of TRPC6 to store depletion in proliferative VSMCs and A7r5 cells. The effect of TRPC6 on coupling of SOCC and NCX has been shown to differ in A7r5 cells and mouse aortic VSMCs [21], and further work is needed to clarify that relationship.

In summary, this study showed that TRPC4–TRPC6 heteromultimerization linked store depletion via STIM1 with NCX reversal in VSMCs.

## Supplementary Material

KCHL_A_1451696.zip
