# Supplementary material for: TRPC6 and TRPC4 Heteromultimerization Mediates Store Depletion-Activated NCX1 Reversal in Proliferative Vascular Smooth Muscle Cells
Source: Channels (Austin). 2018 Apr 10;12(1):119–25. doi: 10.1080/19336950.2018.1451696 (PMC5972809; doi:10.1080/19336950.2018.1451696)
Supplement: KCHL_A_1451696.zip [file kchl-12-01-1451696-s001.zip › KCHL_A_1451696/data supplement.doc]

**Supplementary Methods**

**Reagents**

Thapsigargin (#T9033), KB-R7943 (#K4144) and N-Methyl-D-glucamine (NMDG) (#M2004) were from Sigma (Saint Louis, MO, USA). Fetal bovine serum, medium, and supplements were purchased from Gibco BRL (Billings, MT, USA). Fura-2/AM (#ab120873) was from Abcam (Cambridge, UK). CoroNa™ Green/AM (#c36676) was from Molecular Probes (New York, NY, USA). Lipofectamine^®^ 3000 (#L3000), Opti-MEM^®^ (#31985), Protein A/G Agarose (#20423), HEPES and HBSS were from Life Tech. (New York, NY, USA). Rabbit anti-rat HTRP, mouse anti-rat PCNAwas from Abcam (Cambridge, UK). Rabbit anti-rat STIM1 (#5668) was from Cell Signaling (Danvers, MA, USA). Anti-TRPC1 (#ACC010), anti-TRPC2 (#ACC027), anti-TRPC3 (#ACC016), anti-TRPC4 (#ACC018), anti-TRPC5 (#ACC020), and anti-TRPC6 (#ACC017) in rabbit were from Alomone Lab (Jerusalem, Israel).

**Western blotting**

Vessels or VSMC proteins were extracted as previously described.^1^ Equal amounts of protein were separated by SDS-PAGE (10% and 4–12% polyacrylamide gels) and then transferred onto a polyvinylidene difluoride membrane or nitrocellulose membrane by electroblotting for 4 h at 150 mA. The membrane was blocked in 5% non-fat milk in PBS along with 0.1% Tween 20. The membrane was incubated with primary antibodies, followed by the appropriate secondary antibodies, and detection of speciﬁc proteins was performed by enhanced chemiluminescence following the manufacturer’s instructions. Densitometric signals were quantified by AlphaView SA software.

**Immunofluorescence staining**

Carotid artery sections and coverslip-planted cells were prepared for immunofluorescence staining using a protocol described previously.^2^ The samples were permeabilized with 0.2% Triton X-100 in phosphate-buffered saline (PBS) for 10 min. Endogenous peroxidase activity was quenched and sections were blocked in 10% horse serum in PBS for 1 h. Primary antibody was applied overnight at 4°C and secondary antibody was applied for 1 h at room temperature. Samples incubated with an isotype-matched control antibody were used as negative control. Images were taken with a confocal laser scanning microscope and data were analyzed using Shortcut to Imagel software.

**Cell culture**

Six Sprague-Dawley rats (250–300 g, Mayo Clinic, Rochester, USA) were killed by intraperitoneal injection of an overdose of sodium pentobarbital (100 mg/kg), and these protocols were approved by the Institutional Animal Care and Use Committee at the Mayo Clinic Animal Resource Facility (#A31715). VSMCs from the thoracic aorta of rats were cultured using adherence culture methods. VSMCs were maintained in culture (45% DMEM and 45% Ham’s F10 medium with 10% FBS supplemented with 1% antibiotic) at 37°C, 5% CO_2_ and 100% humidity. Cells were passaged and used within 3–10 passages.^3^

**RNA inference**

To suppress the expression of STIM1, TRPC1, TRPC4, and TRPC6, selected stealth siRNAs were designed according to the rat gene bank (Life Tech.). siRNA sequences are listed in Table 2. Silencer® Select Negative Control was used as the negative control. siRNA was transfected directly into target cells using the Lipofectamine 3000 Transfection reagent following the manufacturer’s instructions. When two siRNA probes were used as a cocktail, each probe was used at half of its usual concentration, and the total siRNA concentration was 1 mol/L.

Table 1 siRNA sequences for rat NCX1, STIM1, TRPC1, TRPC4, and TRPC6

| Target | NCBI UniGene ID | Sequence(5′-3′) | GeneBank accession number |
| --- | --- | --- | --- |
| STIM1 | Rn.106771 | CCAUGACCCUACAGUGAAAtt  UUUCACUGUAGGGUCAUGGta | XM_001069624 |
| TRPC1 | Rn.88592 | CCAUAACUAUUGAAAACGAtt  UCGUUUUCAAUAGUUAUGGta | NM_053558 |
| TRPC4 | Rn.10853 | CGGACACUUUGGGUUUACAtt  UGUAAACCCAAAGUGUCCGta | NM_080396 |
| TRPC6 | Rn.105986 | GUUUCUCCAGAAUAGCUUAtt  UAAGCUAUUCUGGAGAAACtt | NM_053559 |

**Measurement of intracellular free Ca^2+^**

Ca^2+^ measurements were performed as described previously.^2^ VSMCs were loaded with the calcium indicator Fura-2/AM (5 μmol/L) in Tyrode’s solution (in mmol/L: NaCl 145, KCl 4, MgCl_2_ 1, CaCl_2_ 2, HEPES 10, and Glucose 10 at pH 7.4) for 60 min at room temperature. For Ca^2+^ measurements, ﬂuorescent images of several cells were recorded and analyzed with a digital ﬂuorescence imaging system (Nikon Inverted Microscope, TE2000U). Fluorescence emission at 510 nm was monitored using excitation wavelengths of 340 and 380 nm; intracellular Ca^2+^ measurements are shown as 340/380-nm fluorescence ratios. All traces are averages from multiple cells, and are representative of several independent experiments. Ca^2+^-Free Tyrode’s solution (in mmol/L: NaCl 145, KCl 4, MgCl_2_ 7, CaCl_2_ 0, HEPES 10, and Glucose 10 at pH 7.4 with NaOH), Na^+^-Free Tyrode’s solution (in mmol/L: NaCl 0, KCl 4, MgCl_2_ 1, CaCl_2_ 2, HEPES 10, NMDG 145, and Glucose 10 at pH 7.4 with NaOH) and Na^+^/Ca^2+^-Free Herpes-buffered saline (in mmol/L: NaCl 0, KCl 4, MgCl_2_ 7, CaCl_2_ 0, HEPES 10, NMDG 145, and Glucose 10 at pH 7.4 with HCl) were used in our experiments.^4^

**Measurement of intracellular Na^+^ concentration ([Na^+^]i) transients**

[Na^+^]i transients were measured as described previously.^5,6^ Cells were loaded with CoroNa ^TM^ Green-AM (Molecular Probes; 10 μM) for 45 min at room temperature in the loaded solution (in mmol/L: NaCl 145, KCl 4, MgCl_2_ 1, Glucose 10, HEPES 10, and CaCl_2_ 2 at pH 7.4 with NaOH). A linear regression of baseline fluorescence was used to correct raw data, which avoided the error from CoroNa^TM^ Green’s leak out of the cell. As indicated, cells were pre-incubated with KB-R7943 (10 μM) for 10 min prior to stimulation with thapsigargin. Images were acquired with an inverted confocal Laser Scanning Microscope 780 (40× water-dipping). Cells were excited at 490 nm and fluorescence above 515 nm was collected using Zen imaging software (v.2012, Germany). Na^+^-transients were recorded in regions of interest and 2–4 regions different were selected for each cell. Peripheral regions were selected to exclude the nucleus, and central regions included the nucleus. Data are expressed as the fluorescence (F) divided by the baseline fluorescence (F_0_).

**Co-immunoprecipitation**

For immunoprecipitation assays, aliquots of cell lysates were incubated with 2 μg of anti-TRPC4 or anti-TRPC6 antibodies and 20 μl of protein A/G-agarose beads overnight at 4°C on a rocking platform. Proteins in the cell lysates or immunoprecipitated samples were separated on 4%–12% SDS-PAGE gels, transferred onto nitrocellulose membranes, and the membranes were incubated with primary antibodies, followed by secondary antibodies. Detection of speciﬁc proteins was performed by enhanced chemiluminescence following the manufacturer’s instructions. Densitometric signals were quantified by AlphaView SA software.^7^

**Statistical analysis**

Results are expressed as the mean ± SEM. SPSS software (v.19.0 IL, USA) was used for statistical analysis. Student’s *t* tests were used to compare two groups, and ANOVA was used with Tukey’s multiple comparison tests for multiple groups. Values of *P* <0.05 were regarded as statistically significant.

**Supplementary References**

1. Guo RW, Yang LX, Wang H, Liu B, Wang L. Angiotensin II induces matrix metalloproteinase-9 expression via a nuclear factor-kappaB-dependent pathway in vascular smooth muscle cells. *Regul Pept*. 2008;147:37-44.

2. Guo RW, Wang H, Gao P, Li MQ, Zeng CY, Yu Y, Chen JF, Song MB, Shi YK, Huang L. An essential role for stromal interaction molecule 1 in neointima formation following arterial injury. *Cardiovasc Res*. 2009;81:660-668.

3. Guo RW, Yang LX, Li MQ, Pan XH, Liu B, Deng YL. Stim1- and Orai1-mediated store-operated calcium entry is critical for angiotensin II-induced vascular smooth muscle cell proliferation. *Cardiovasc Res*. 2012;93:360-370.

4. Sathish V, Delmotte PF, Thompson MA, Pabelick CM, Sieck GC, Prakash YS. Sodium-calcium exchange in intracellular calcium handling of human airway smooth muscle. *PLoS One*. 2011;6:e23662.

5. Poburko D, Liao CH, Lemos VS, Lin E, Maruyama Y, Cole WC, van Breemen C. Transient receptor potential channel 6-mediated, localized cytosolic [Na^+^] transients drive Na^+^/Ca^2+^ exchanger-mediated Ca^2+^ entry in purinergically stimulated aorta smooth muscle cells. *Circ Res*. 2007;101:1030-1038.

6. Meier SD, Kovalchuk Y, Rose CR. Properties of the new fluorescent Na^+^ indicator CoroNa Green: comparison with SBFI and confocal Na^+^ imaging. *J Neurosci Methods*. 2006;155:251-259.

7. Wu G, Cai J, Han Y, Chen J, Huang ZP, Chen C, Cai Y, Huang H, Yang Y, Liu Y, Xu Z, He D, Zhang X, Hu X, Pinello L, Zhong D, He F, Yuan GC, Wang DZ, Zeng C. LincRNA-p21 regulates neointima formation, vascular smooth muscle cell proliferation, apoptosis, and atherosclerosis by enhancing p53 activity. *Circulation*. 2014;130:1452-1465.
